# Efficient and crucial quality control of HAP1 cell ploidy status

**DOI:** 10.1242/bio.057174

**Published:** 2020-11-12

**Authors:** Tobias B. Beigl, Ine Kjosås, Emilie Seljeseth, Nina Glomnes, Henriette Aksnes

**Affiliations:** 1 Department of Biomedicine, University of Bergen, 5020 Bergen, Norway; 2 Institute of cell biology and immunology, University of Stuttgart, D-70569 Stuttgart, Germany; 3 Department of Biological Sciences, University of Bergen, 5020 Bergen, Norway; 4 Department of Clinical Science, University of Bergen, 5020 Bergen, Norway

**Keywords:** HAP1, Near-haploid human cell line, CRISPR/Cas9, Gene editing, clustered palindromic repeats, Cas9 enzyme, Cell phenotype analyses, Microscopy genetic disease cell model, Cell culture quality control

## Abstract

The near-haploid human cell line HAP1 recently became a popular subject for CRISPR/Cas9 editing, since only one allele requires modification. Through the gene-editing service at Horizon Discovery, there are at present more than 7500 edited cell lines available and the number continuously increases. The haploid nature of HAP1 is unstable as cultures become diploid with time. Here, we demonstrated some fundamental differences between haploid and diploid HAP1 cells, hence underlining the need for taking control over ploidy status in HAP1 cultures prior to phenotyping. Consequently, we optimized a procedure to determine the ploidy of HAP1 by flow cytometry in order to obtain diploid cultures and avoid ploidy status as an interfering variable in experiments. Furthermore, in order to facilitate this quality control, we validated a size-based cell sorting procedure to obtain the diploid culture more rapidly. Hence, we provide here two streamlined protocols for quality controlling the ploidy of HAP1 cells and document their validity and necessity.

This article has an associated First Person interview with the co-first authors of the paper.

## Abstract (precis)

**Summary:** Sharing an effective procedure to quality control the near-haploid HAP1 cells for standardized comparison to CRISPR/Cas9 modified versions and demonstrating the need for controlling the spontaneous diploidization of HAP1 cultures.

## INTRODUCTION

Mammalian somatic cells are usually diploid, containing one genome copy from each parent, while haploidy is typically confined to the germline (Wutz, 2014). However, aberrant chromosome loss may occur during tumorigenesis (Zasadil et al., 2013) and rare human cancers with a hypodiploid karyotype have been found, for example in leukemia (Oshimura et al., 1977; Andersson et al., 1995). HAP1 is a near-haploid adherent fibroblast-like cell line of human leukemia origin. Such haploid cells are powerful tools for studying gene function as they contain a single copy of the genome and are thus unable to mask the effect of any mutations, which could possibly occur in +/− heterozygotes (Li and Shuai, 2017). The HAP1 cell line has therefore recently become a very popular subject for CRISPR/Cas9 editing since, for most genes, the mutation of a single allele is sufficient to reveal a potential phenotype. Through the gene-editing company Horizon Discovery, there are at present as many as 7500 edited cell lines available and more are continuously generated. Hence, such rare haploid human cells are very valuable as they allow human cell cultures to get a taste of the ‘awesome power of yeast genetics’ (Stark, 2001; Cavanaugh and Jaspersen, 2017).

The HAP1 cell line is derived from another near-haploid cell line KBM7, originating from a male with chronic myelogenous leukemia. More specifically, HAP1 is a subclone of the KBM7, which is haploid except for a disomy of chromosome 8 (25, XY, +8, Ph+) (Kotecki et al., 1999). HAP1 was obtained in an attempt to generate induced pluripotent stem cells from KBM7 cells by expression of OCT4, SOX-2, c-MYC and KLF411 (Carette et al., 2011). As opposed to KBM7, the subclone denoted HAP1, grew adherently and did not express hematopoietic markers. Carette et al. (2011) described the majority of these HAP1 cells in early-passage cultures to be haploid for all chromosomes, including chromosome 8, which is diploid in KBM7 cells. The HAP1 cells used as parental cells by Horizon Discovery have a single copy of all chromosomes, except a portion of chromosome 15. Later on, HAP1 cells were engineered by a CRISPR/Cas9-based chromosome-scale genome engineering strategy to become fully haploid and were designated eHAP (Essletzbichler et al., 2014). However, the original HAP1 cells are the basis for the large gene-knockout library existing today.

Ploidy instability has for long been recognized as a typical trait of haploid cultures. For HAP1 cells a rapid reduction in the percentage of haploid cells has been described (Olbrich et al., 2017), consistent with observations from other haploid human cells (Yilmaz et al., 2016). The loss of HAP1 haploidy has been connected to uncoordinated centrosome cycle, causing diploid cells to occur in the culture (Yaguchi et al., 2018). It was also recently shown that loss of the haploid state is due to overgrowth by diploid cells present in the cultures, as haploid cells are limited in their viability due to activation of a p53-dependent response (Olbrich et al., 2017). Furthermore, to facilitate maintenance of the valuable haploid cells, single-cell subcloned haploid HAP1 cell lines were shown to be more ploidy-stable (Olbrich et al., 2017). Additionally, a chemical screen identified compounds capable of selecting for haploidy (Olbrich et al., 2019).

Although the haploid state is critical for the success of genetic screens, it is not a necessity for more cell-biology-focused studies. Given the great potential of genetically modified HAP1 cells as a tool to study cellular phenotypes of gene knockouts, it is a concern that differing ploidy states between cultures of parental control and knockout cells may constitute a source of interfering variables. The main aim of this article is to check for such potential fundamental differences between HAP1 cell cultures in the haploid versus diploid state. We indeed reveal such differences and argue the importance of ploidy-controlling HAP1 cells prior to phenotypic analyses, such as, for example, for studies of the intracellular organization of organelles (Capmany et al., 2019a; Latge and Schauer, 2019) or cytoskeletal morphology (Johnsson and Karlsson, 2012; Yang et al., 2017; Capmany et al., 2019b). Importantly, we present effective, affordable and streamlined procedures to obtain diploid cultures.

## RESULTS AND DISCUSSION

### Flow cytometry revealed transition from haploid to diploid cultures of HAP1 cells

**Fig. 1.**
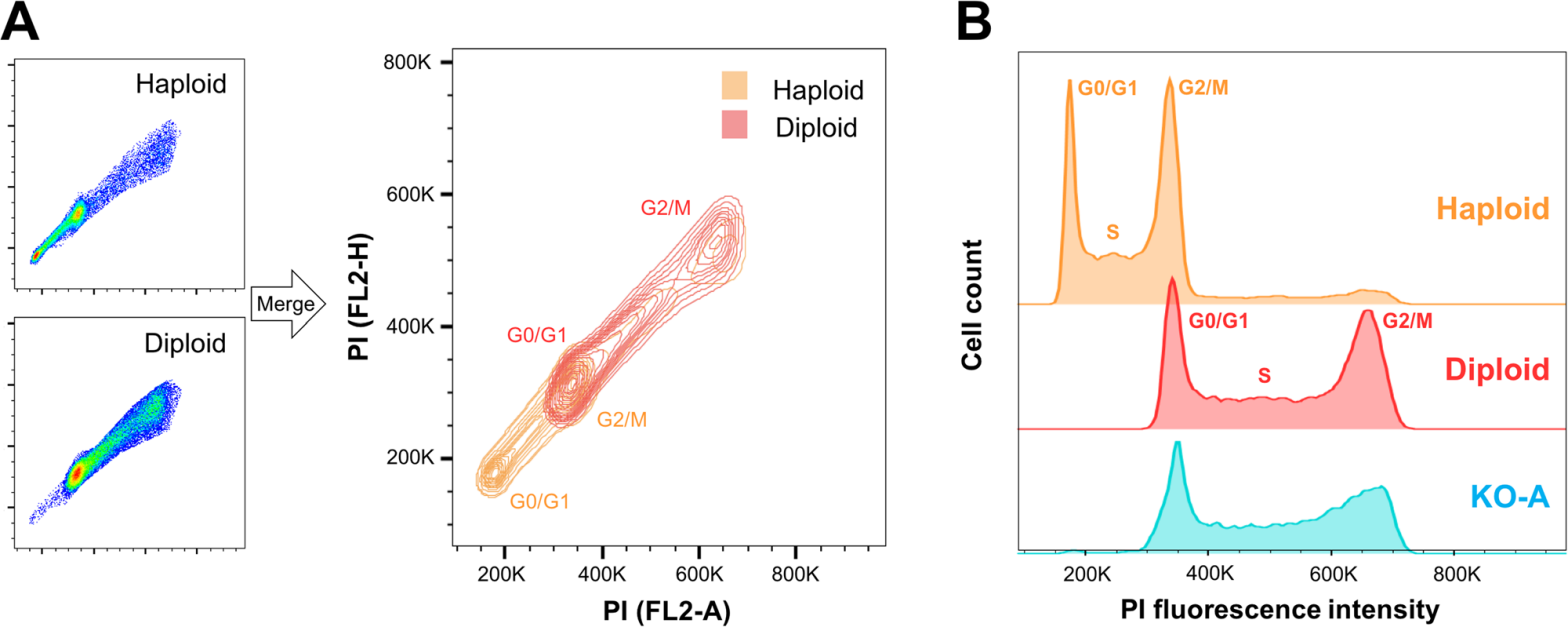
**Haploid and diploid HAP1 cells can be distinguished based on PI stain in a flow cytometer.** (A) Ploidy-verified reference cells (HAP1 C631 haploid and diploid) were fixed, DNA-stained with PI and analyzed on a BD Accuri C6. Linear plots of fluorescence intensity height (FL2-H) against total cell fluorescence (FL2-A) illustrate the difference in DNA-quantity between haploid (orange) and diploid (red) HAP1 cells. (B) Example result of ploidy test showing flow data for the cell line ‘Gene specific KO-A’ at passage 28, referenced to ploidy-verified control lines. Histograms show number of cells against intensity of the DNA. Fluorescence intensity is shown in linear scale (both A and B).

Although most commonly used in cell-cycle analysis (Pozarowski and Darzynkiewicz, 2004; Kim and Sederstrom, 2015; Li, 2017; Rosebrock, 2017), flow cytometry is a verified method to determine ploidy (Rickes et al., 2003; Darzynkiewicz et al., 2017; Todd et al., 2018). Aiming to verify ploidy status of various passages of HAP1 cells, we measured several different HAP1 knockout cells along with haploid and diploid reference cells. We first validated flow cytometry on propidium iodide (PI)-stained cells as an effective method to assess the ploidy (Fig. 1A). Here we initially used low passage HAP1 from Horizon as well as a diploid HAP1 clone obtained from Horizon for reference. The difference in DNA-quantity between haploid (orange) and diploid (red) HAP1 cells, demonstrate that ploidy status can be determined with PI-based flow cytometry. For each of the reference lines, two main populations were observed (Fig. 1A, left). These represent the different cell-cycle stages, G0/G1 (lower left cluster) and G2/M (upper right cluster), since the latter contain twice the amount of DNA as these cells have passed S-phase. As expected, overlaying the haploid and diploid plots (Fig. 1A, right), revealed an overlap between the DNA content of haploid cells in the G2/M phase and diploid cells in the G0/G1 phase.

Next, we analyzed several gene-specific knockout cells (denoted KO-A, KO-B, etc.) at various passages (example shown in Fig. 1B). In this plot, the cell phases G0/G1 and G2/M cause two peaks. Again, the double amount of DNA in G2/M phase reason an overlap between fluorescence intensity of the DNA stain for haploids in G2/M and diploids in G0/G1. The gene-specific knockout (KO-A) that was tested here at passage 28, matched the diploid reference line. We observed a tendency of the near-haploid HAP1 cells to become diploid during normal cultivation. This did however not affect the gene knockout (Drazic et al., 2018; Beigl et al., 2020, Ree et al., 2020). We found that the HAP1 cell cultures typically became diploid within a relative short time-frame, typically around passage 20 post CRISPR (Table 1). Once diploid, the ploidy number was stable. Furthermore, in cultures tested between passage 7 and 20, there were typically some diploid cells present. Based on this, we concluded that HAP1 cells typically start to turn diploid around passage 10 and most likely reach a pure diploid state around passage 20–30. Although it was shown previously that the ploidy instability is mainly caused by a growth advantage of the diploid over haploid HAP1 cells (Olbrich et al., 2017), genetically modified cell lines are subcloned following the CRISPR/Cas9, meaning that these originate from a single haploid cell. Hence, true diploidization, entailing conversion of a haploid cell into a diploid, is likely to be underlying the ploidy instability observed here. Notably, knockdown was stable after diploidy was confirmed (Drazic et al., 2018; Oishi et al., 2018), meaning that knockout cells transitioning into diploids should represent −/− homozygotes.

**Table 1.**
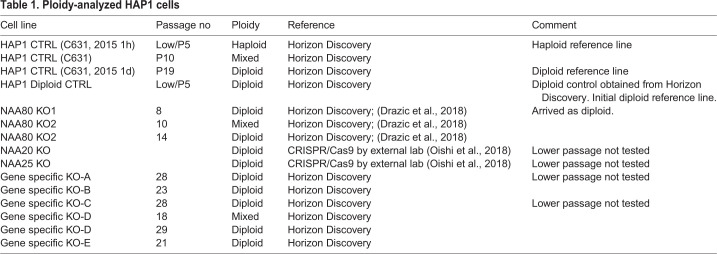
**Ploidy-analyzed HAP1 cells**

### Haploid and diploid HAP1 cells are fundamentally different

**Fig. 2.**
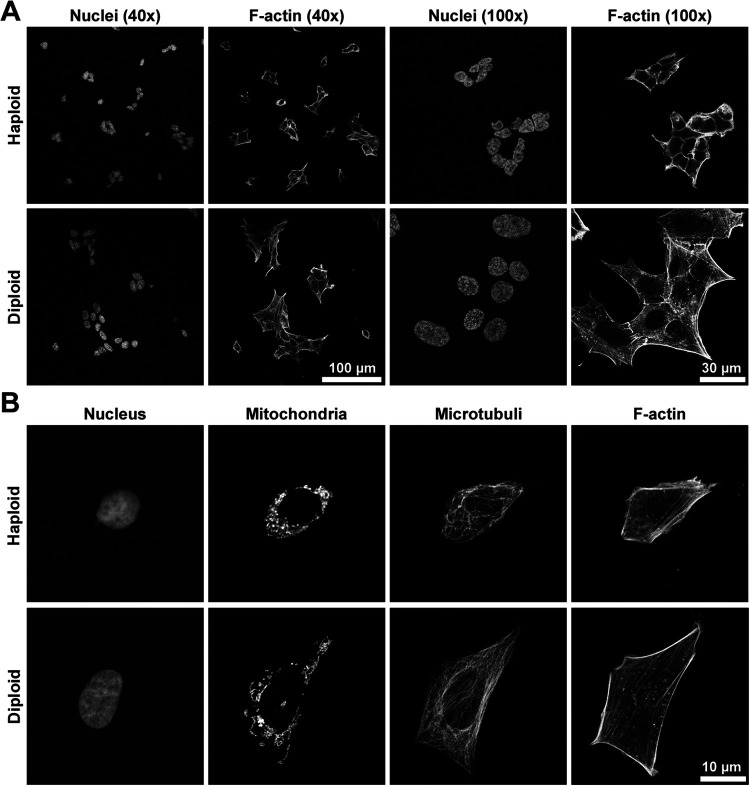
**Confocal imaging of haploid and diploid HAP1 cells showed large ploidy-dependent differences.** Ploidy-verified haploid and diploid HAP1 cells subjected to immunocytochemistry and imaged with a confocal microscope. (A) Overview of cell culture as observed with 40× objective (left) and 100×objective (right). Nuclei were visualized with DAPI and F-actin with fluorescent phalloidin. (B) A closer look at structures in single cells (3×optical zoom, 100×objective). Nuclei and F-actin were stained as in A. In addition, mitochondria were visualized with antibody towards Cox IV and microtubules with antibody towards β-tubulin.

After confirming that HAP1 cells did in fact turn diploid over time/passages, we performed some basic characterizations to compare ploidy-verified low-passage HAP1 haploids to their higher-passage, diploidized versions. These analyses revealed several fundamental differences. As apparent in Fig. 2, immunocytochemistry analysis of fixed cells revealed drastic differences in 2D cell size, including nucleus size. Nuclear shape was typically more oval for the diploids and they were more spaced from one another (Fig. 2A). Investigations at higher magnification revealed that there was a large ploidy-dependent difference in the appearance of the subcellular structures mitochondria, microtubules and microfilaments (Fig. 2B). Hence, upon analysis of subcellular structures with fluorescence microscopy we found drastic differences in the appearance of haploid versus diploid HAP1 control cells. This points to the necessity of performing ploidy-controlling experiments prior to phenotyping experiments in order to avoid ploidy as a confounding variable. Furthermore, it was demonstrated here that diploid cultures were far better suited to this type of microscopy due to their larger size and more spread-out nature on coverslips.

**Fig. 3.**
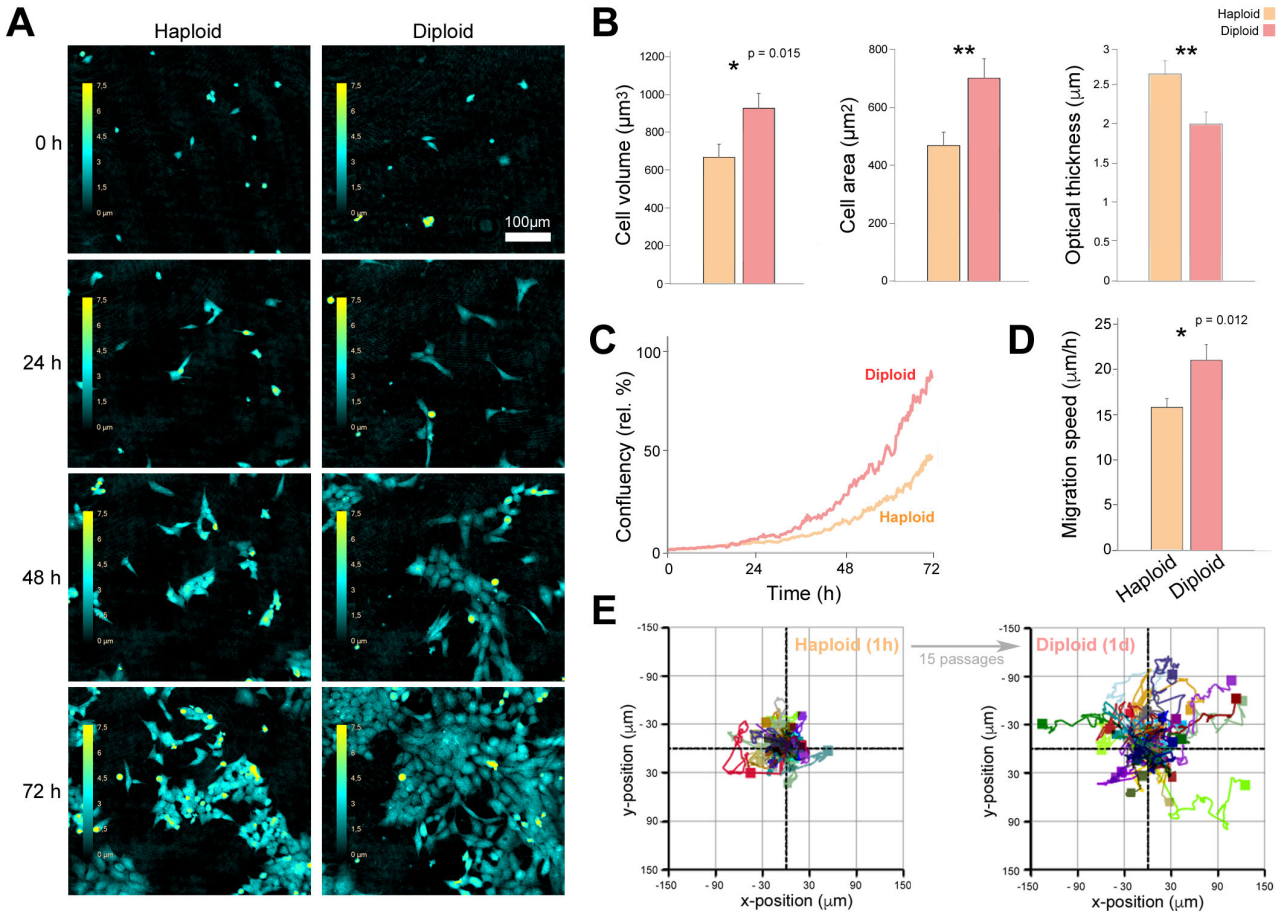
**Live-cell phase holographic imaging analysis of haploid and diploid HAP1 cells show basic and specific ploidy-dependent differences.** Ploidy-verified low-passage HAP1 haploids (2015 batch of C631, 1h) and their higher-passage diploid versions (1d) were seeded on laminin and monitored in a HoloMonitor M4 for 72 h with image acquisition every 15 min (A–E). (A) 2D representation of 3D holographic images at selected time points. The color bar indicates optical thickness or cell height information (z-values) for these 2D projected 3D images ranging from 0 µm (black) to 7.5 µm (yellow). Scale bar, 100 µm. (B) Morphological cell properties measured based on holographic images at 24 h (cell volume) or 48 h (cell area and optical thickness) post seeding. (C) Cell growth based on confluency measure of cell-covered area, relative to t=0. (D) Migration speed of random single-cell migration in culture measured from 24 to 48 h post seeding from two independent wells of haploid 1h (*n*=38) and diploid 1d (*n*=40) HAP1 cells. (E) Single-cell trajectory rose plots of the same cells as in D. For all bar charts, * *P*<0.05 and ** *P*<0.005 as determined with *t*-test (unpaired, two-tailed with Welch's correction) and error bars represent s.e.m. (only upper part shown).

The same ploidy-verified low-passage HAP1 haploids and their corresponding higher-passage diploids were also observed with a live-cell imaging device using phase holographic imaging. The different appearance of haploid and diploid HAP1s was also visible here (Fig. 3A). Diploids were larger, as observed previously, and also flatter. These observations were confirmed with quantification of several cell parameters measured based on the holographic three-dimensional (3D) images. Increased cell size of the diploid was detected for both 3D (cell volume) and 2D (cell area). In addition, haploids had a higher optical thickness, confirming the diploid's tendency to spread to a flatter morphology on the coverslip. In agreement with previous reports, the diploid increased its confluency more rapidly (Fig. 3C), indicating a capacity to outgrow the haploids, as previously reported (Olbrich et al., 2017). With these basic ploidy-dependent differences, we were curious as to whether further cell properties could be altered in diploidized HAP1s. A consistent observation following diploidization of cultures was the tendency for less colony-like tight packing, but rather more separation of cells. We therefore suspected increased random motility of the diploid and conducted a single-cell tracking to characterize the motility properties, similar to previous phenotypic analyses (Aksnes et al., 2018). Indeed, the diploids were here indicated to have an increased motility compared the haploids (Fig. 3D and E). Overall, our findings in Figs 2 and 3 clearly demonstrate that diploidized HAP1 cells have specific differences compared to haploid HAP1 cells, hence underscoring how crucial it is to control for ploidy before embarking on phenotypic analyses.

### Fast forwarding to the diploid state by means of size-based cell sorting

**Fig. 4.**
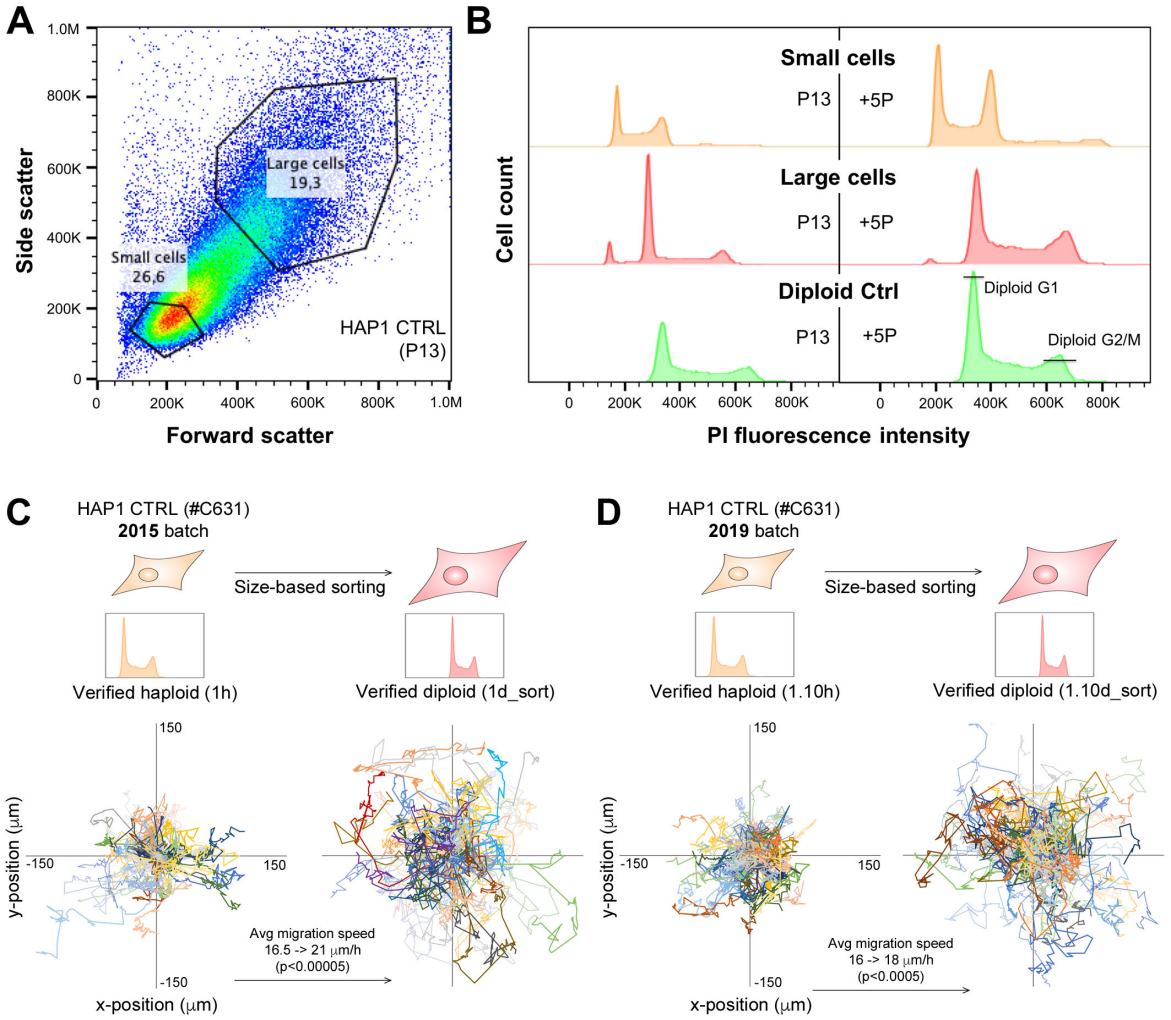
**Haploid and diploid HAP1 cells can be separated based on size in a cell sorter.** HAP1 parental control cell line (C631, 2015 batch) was cultured until passage 13 (P13), at which point small and large cells were divided into separate cultures on a Sony SH800 cell sorter (A,B). (A) Gating strategy for sorting of small (putative haploid) and large (putative diploid) cells. (B) Outputs from the size-based cell sorting were analyzed for ploidy determination by flow cytometry on fixed PI-stained cells 1 week after sorting (left) and five passages later (right). (C,D) HAP1 control (CTRL) and its diploid version obtained by size-based cell sorting were seeded for live-cell holographic imaging followed by single-cell migration analysis as in Fig. 3D,E. Two different original batches of HAP1 CTRL (C631) were used (C, 2015 batch; and D, 2019 batch) and these were individually subjected to the size-based cell sorting strategy to isolate diploid cells. Tracking data from two independent experimental setups each analyzing all four cell lines from three different wells (six in total) are shown merged. *P*<0.00005 (C) and *P*<0.0005 (D) was determined with *t*-test (unpaired, two-tailed with Welch's correction), x- and y-axis scales are identical for all four rose plots in C and D.

Obtaining diploid cultures by continued culturing of HAP1 cells for 15–30 passages takes time and also entails a risk of contamination, random genetic alterations and long-term phenotype recovery. We therefore wanted to optimize the routines in order to speed up this process and thus be able to obtain diploid cultures of lower passage. Here, we made use of the difference in cell size observed between haploid and diploid HAP1 parental control cells (Figs 2 and 3), and performed a size-based cell sorting using a Sony SH800 flow cytometer. As shown above, mere passaging caused HAP1 cells to start turning diploid around passage 10. Therefore, we hypothesized that sorting cells in their early mixed phase could enrich for the diploid cells and remove haploid cells. During sorting of cells (Fig. 4A), it was possible to distinguish a small cell population from a large cell population and using the indicated gating strategy, these were separated into different tubes. Next, we analyzed both output cultures for ploidy along with an already confirmed diploid control (Fig. 4B). The analysis showed a more or less pure haploid population for the small cells, and a near pure diploid population for the large cells. After five passages post sorting, the population of small cells started to turn diploid, while importantly the population of large cells was nearly pure diploid. Furthermore, we observed that the diploid reference line was stable diploid at higher passage. We concluded that using this size-based cell sorting strategy one can obtain a nearly pure diploid cell population at passage 18 and possibly earlier. However, a limitation of size-based cell sorting is that it may not be ideal in cases where cell size is a phenotypic trait of the knockout.

Next, we wanted to strengthen our observation above indicating that diploid cells have increased migration. Here, we performed a migration-tracking assay on diploids obtained by size-based sorting, in order to investigate whether we would observe the same effect as for the diploids obtained by cultivation time/passaging (Fig. 3D and E). We used two different batches of HAP1 Ctrl cells (#C631), one obtained as haploid in 2015 (used in Figs 2 and 3) and one obtained as haploid in 2019. These were independently subjected to diploid enrichment by size-based cell sorting (as in Fig. 4A and B). The two sets of haploids and diploids (2015 batch in Fig. 4C; 2019 batch in Fig. 4D) showed an increased migration speed for the diploids, and therefore validated our initial finding from the diploids obtained after longer cultivation/passaging. Note that in order to ensure purest possible ploidy, the haploid was analyzed at a very low passage (a maximum of five in-house passages). Therefore, although size-based sorting reduces the time in culture for the diploid, there is some difference in the time spent in culture in-house, and hence it cannot be completely ruled out that this may have contributed to the increased migration of the diploid.

## CONCLUSION

**Fig. 5.**
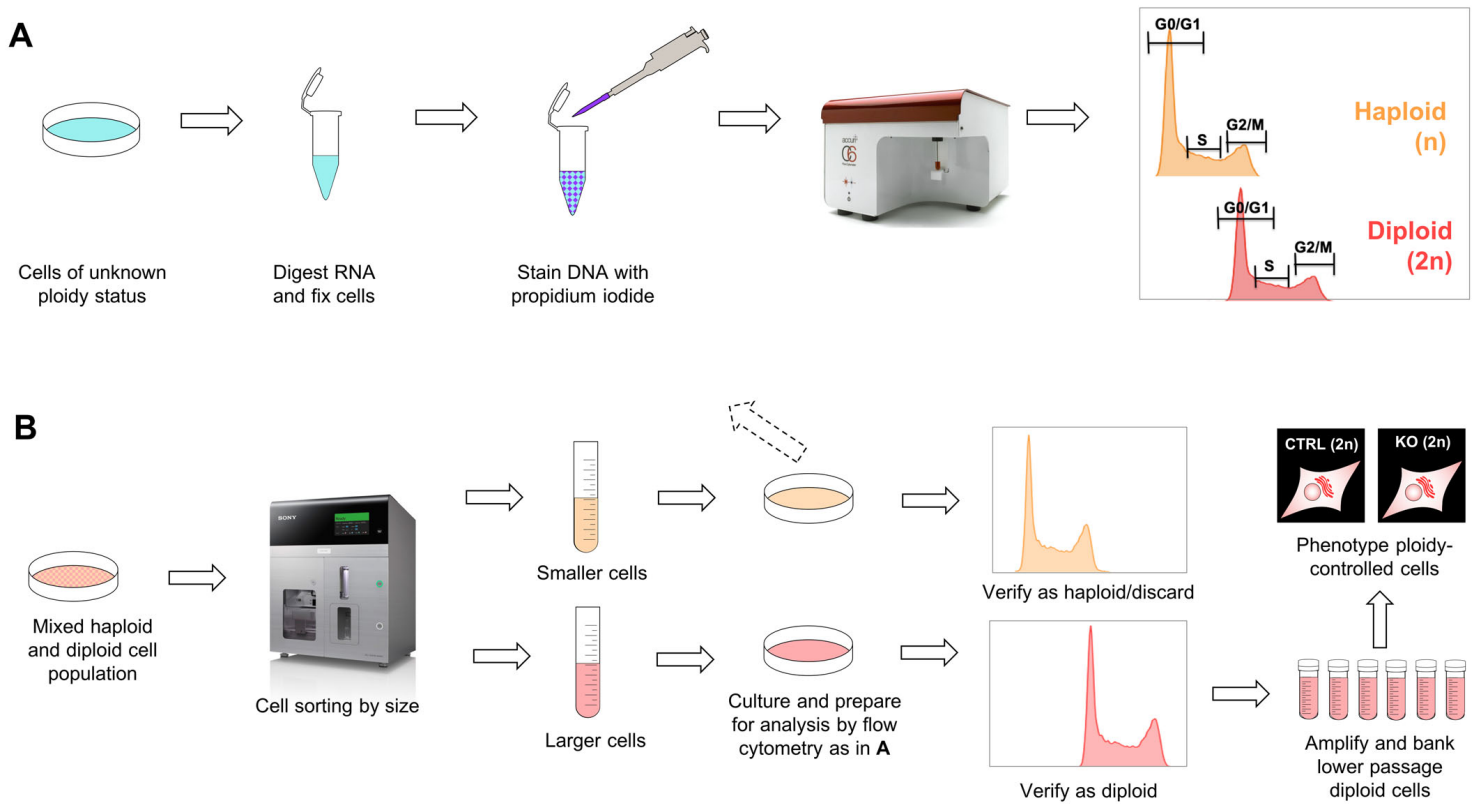
**Schematic overview of workflow to obtain diploid cultures of HAP1.** (A) Method of distinguishing haploid and diploid HAP1 cells by means of flow cytometry on PI-stained cells. (B) Method to fast-forward to diploid cultures by means of size-based cell sorting. The method in B can also be used to select for haploidy.

Near-haploid human cell lines are instrumental for genome engineering since gene knockout is eased by the absence of a second gene copy. However, once the gene-specific knockouts are achieved, and phenotypic analyses of knockout cells are to be compared to parental control cells, it is of great importance to be aware of ploidy instability as a potential source of interfering variables. We reported here that HAP1 cells spontaneously switch to a diploid state during normal cultivation. Importantly, we showed that haploid and diploid cultures are fundamentally different. As diploid cells are still homozygotic for the genetic alteration, in addition to being better suited for cell biological analysis like microscopy, we argue that HAP1 cell lines should either be passaged until diploidy is reached or subjected to size-based cell sorting to obtain diploidy. We presented here effective, streamlined procedures to rapidly obtain diploid HAP1 cells. This procedure is summarized in Fig. 5. Our findings should facilitate the correct use of genetically modified HAP1 cells in cell biological research, and thus further enhance the impact of this powerful tool beyond genetic screens and into the more detailed phenotypic analyses.

## MATERIALS AND METHODS

### Cell culture

HAP1 cells were cultured as recommended by Horizon Discovery. Culture medium was IMDM supplemented with 10% FBS and 1% Penicillin/Streptomycin. Cells were maintained in an incubator with 37°C, 5% CO_2_ and humidified atmosphere, and passaged approximately every 2–3 days. HAP1 cells used in this study were two different batches, 2015-batch (internal number 1h) and 2019-batch (internal number 1.10h), of the parental control (Horizon Discovery, Ltd. catalogue number C631), and their diploid derivatives obtained either by cultivation/passaging in our lab (1d) or size-based cell sorting as described herein (1d_sort and 1.10d_sort). In addition, a HAP1-diploid control originally sent from Horizon Discovery upon request (catalogue number not available) was used initially as a positive control to confirm the diploidization tendency. After confirmation of the diploidization tendency, several gene-specific HAP1-knockout cells were also passaged and subjected to ploidy determination and the passage number of obtained diploid was noted (overviewed in Table 1, but otherwise not used in this work).

### Determination of ploidy status by flow cytometry

A subconfluent (70–80%) 6 cm dish was harvested by trypsinization. A microscope was used to ensure that cells were properly singularized. Cells were counted and 2×10^6^ cells were pellet by centrifugation at 300× ***g*** for 5 min at RT. Pellets were washed twice with 1 ml PBS (and transferred to an Eppendof tube). Cells were fixed in 500 µl ice cold 70% EtOH as follows: first pellets were thoroughly resuspended in 150 µl PBS, and thereafter 350 µl EtOH (-20°C) was added drop-by-drop while vortexing at slow speed (1400) and incubated at -20°C for at least 1 hour. On the day of flow analysis, pellet was washed by adding 800 µl cold PBS before centrifugation at 300× ***g***, for 5 min. Wash was repeated once in 1 ml PBS followed by centrifugation. Cell pellets were then resuspended in 500 µl PBS containing 200 μg/ml heat-treated RNaseA 17,500 U (Qiagen, 19101) and incubated at 37°C, 30 min. Samples were flushed through a Falcon 5 ml polystyrene cell-strainer-capped tube (ref 352235, VWR 734-0001) to ensure single cells before propidium iodide was added to a final concentration of 50 µg/ml.

### Assessing ploidy status by flow analysis

Flow cytometry analysis of PI-stained cells was performed with a BD Accuri C6. The limit was set to 10,000 cells and fluidics speed was set to fast. Cells of known ploidy were used as controls each time. Ploidy was determined based on plots showing cell count against the fluorescence intensity of PI, as the haploid cells in the G2/M phase overlap with the diploid cells in the G0/G1 phase. Data was processed and visualized using FlowJo.

### Immunofluorescence staining and microscopy

Ploidy-verified haploid (C631, 2015, 1h) and diploid (C631, 2015, 1d) HAP1 control cells were seeded on #1.5H glass coverslips approximately 24 h prior to fixation. Cells were fixed in 3% PFA in phosphate buffer (0.056 M NaH_2_PO_4_ +0.144 M Na_2_HPO_4_). Subsequent to incubation for 25 min at RT, cells were washed three times with PBS and permeabilized using 0.1% Triton™ X-100 with incubation for 10 min at RT. Samples were washed again three times with PBS. Next, samples were incubated with blocking solution (10% BSA+1% goat/donkey serum in PBS) for 1 hour at room temperature (RT) on a shaker with gentle tilting. The primary antibodies were applied at 1:200, diluted in PBS with 2% BSA and 2% goat or donkey serum. Prior to use, any formed antibody aggregates were centrifuged down (3 min, 16,000× ***g***). Primary antibodies used were rabbit-anti-CoxIV (Cell Signalling Technologies, 4850) and mouse-anti-β-tubulin (Sigma-Aldrich, T5293). Coverslips were incubated for at least 1 hour, cell-side down, on drops of the antibody solution in a dark humidity chamber at RT. Afterwards, coverslips were washed three times with PBS and left in the third washing step for at least 1 hour with low-speed tilting. Secondary antibodies with conjugated fluorophores were used as above, except at 1:100. Phalloidin-Atto 647N (Sigma-Aldrich, 65906) was used in the same staining step in a 1:50 dilution. Samples were continuously washed on a gentle rocker for at least 1 hour at RT (or at 4°C, over night). For the final mounting step, the coverslips were dipped twice in MilliQ water and after carefully removing excessive water, mounted on drops of ProLong Diamond Antifade Mountant with DAPI. Mounted coverslips were left to dry in the dark at RT over night.

Confocal (STED) images were obtained using a Leica TCS SP8 STED 3× confocal laser microscope equipped with a HC PL APO STED 100×1.4 NA oil objective, 1 Airy unit pinhole aperture and the appropriate filter combinations. The used lasers were a 405-nm blue-diode (50 mW), white-light (470–670 nm lambda range, power ∼1.5 mW per line, pulsed supercontinuum), and a 775 nm depletion (STED) laser. Images were acquired with the Leica Application Suite X software, exported and processed in ImageJ.

### Live-cell holographic imaging with HoloMonitor M4

Phase holographic imaging of live unlabeled cells was performed with the digital phase holographic imager HoloMonitor M4, as previously (Aksnes et al., 2018, Zhang et al., 2020). For experiments in Fig. 3, ploidy-verified haploid (C631, 2015, 1h) and diploid (C631, 2015, 1d) HAP1 CTRL cells were seeded in µ-dishes 35 mm high (ibidi 81156) precoated with laminin (11 µg/ml, 2 h). 50,000 cells were seeded per plate in 3 ml cell culture medium. The cells were incubated for 20 min at RT during initial cell attachment, before HoloLids were placed on each dish ensuring optimized imaging. Cells were imaged every 15 min with at least two different fields of view per dish. The holographic images were analyzed with HStudio software. Single cells were detected using the Auto-Otsu setting for background and minimum object size was set to 25. Based on the 3D holographic information, several cell morphological parameters were quantitatively measured. For the two independent experimental replicates in Fig. 4C and D, cells were seeded on laminin-coated wells in a µ-plate 24-well black (ibidi 82406). For experiments in Fig. 4C, ploidy-verified haploid (C631, 2015, 1h, internal passage 5/6) and diploid (C631, 2015, 1d_sort, internal passage 18/19) HAP1 control cells were used, and for experiments in Fig. 4D, ploidy-verified haploid (C631, 2019, 1.10h, passage 9/10) and diploid (C631, 2019, 1.10d_sort, passage 17/18) HAP1 control cells were used. Imaging and analysis was performed as above, except three fields from each of three different wells were imaged per cell line.

### Size-based sorting of haploid and diploid cells from mixed cultures

To prepare HAP1 cells for sorting, one 10 cm dish at 70–80% confluency was trypsinized, washed twice in PBS and resuspended in 500 µl culture medium. For sorting of HAP1 cells based on size, a Sony SH800 high-speed multilaser flow cytometer and cell sorter was used. Cell suspensions were run with a sample pressure between 1 and 4 (event rate 1000–1500 events/second). Cells were detected using the 488 nm laser. Sensor gain: FSC, 2 or 3; BSC, 31–33%. Cells were sorted using semi-purity mode. Sort efficiency of 90% or higher was preferred. 100,000 cells was the basis for the plot and cells/events was adjusted at approximately half completion. A size-based gating strategy was used (Fig. 4).

Sorted cells (small: putative haploid and large: putative diploid) were collected in cell culture medium and seeded in appropriately sized dishes depending on the number of cells in the output. Medium was changed approximately 1 hour after seeding.
